# Umbilical cord mesenchyme stem cell local intramuscular injection for treatment of uterine niche: Protocol for a prospective, randomized, double-blinded, placebo-controlled clinical trial

**DOI:** 10.1097/MD.0000000000008480

**Published:** 2017-11-03

**Authors:** Dazhi Fan, Shuzhen Wu, Shaoxin Ye, Wen Wang, Xiaoling Guo, Zhengping Liu

**Affiliations:** a Foshan Institute of Fetal Medicine; b Department of Obstetrics, Southern Medical University Affiliated Maternal & Child Health Hospital of Foshan, Foshan, Guangdong; c Department of Epidemiology and Biostatistics, School of Public Health, Anhui Medical University, Hefei, Anhui, China.

**Keywords:** mesenchyme stem cells, protocol, RCT, uterine niche

## Abstract

**Background:**

Uterine niche is defined as a triangular anechoic structure at the site of the scar or a gap in the myometrium at the site of a previous caesarean section. The main clinical manifestations are postmenstrual spotting and intrauterine infection, which may seriously affect the daily life of nonpregnant women. Trials have shown an excellent safety and efficacy for the potential of mesenchymal stem cells (MSCs) as a therapeutic option for scar reconstruction. Therefore, this study is designed to investigate the safety and efficacy of using MSCs in the treatment for the uterine niche.

**Methods/design:**

This phase II clinical trial is a single-center, prospective, randomized, double-blind, placebo-controlled with 2 arms. One hundred twenty primiparous participants will be randomly (1:1 ratio) assigned to receive direct intramuscular injection of MSCs (a dose of 1∗10^7^ cells in 1 mL of 0.9% saline) (MSCs group) or an identical-appearing 1 mL of 0.9% saline (placebo-controlled group) near the uterine incision. The primary outcome of this trial is to evaluate the proportion of participants at 6 months who is found uterine niche in the uterus by transvaginal utrasonography. Adverse events will be documented in a case report form. The study will be conducted at the Department of Obstetric of Southern Medical University Affiliated Maternal & Child Health Hospital of Foshan.

**Discussion:**

This trial is the first investigation of the potential for therapeutic use of MSCs for the management of uterine niche after cesarean delivery.

**Conclusion:**

This protocol will help to determine the efficacy and safety of MSCs treatment in uterine niche and bridge the gap with regards to the current preclinical and clinical evidence.

**Trial registration number:**

NCT02968459 (Clinical Trials.gov: http://clinicaltrials.gov/).

## 1 Introduction

Approximately 210 million women become pregnant annually, and about 140 million newborn babies are born each year around the world.^[1]^ Of these infants, about one-third are delivered by cesarean section in the United States.^[2,3]^ In China, the overall annual rate of cesarean deliveries increased from 28.8% in 2008 to 34.9% in 2014, a mean increase of 1.0% point per year.^[4]^ Although it allows safe in many situations, the risks of severe maternal complications associated with cesarean delivery are higher than those associated with vaginal delivery.^[5]^ These maternal complications include short-term, such as hemorrhage, anesthetic complications, cardiac arrest, wound hematoma, puerperal infection, venous thromboembolism,^[6]^ long-term, such as chronic pelvic pain, pelvic adhesions, infertility, ectopic pregnancy,^[7]^ the next pregnancy, such as scar pregnancy with life-threatening bleeding, spontaneous abortion, preterm birth, small for gestational age fetus, morbidly adherent placenta, dehiscence or uterine rupture,^[8,9]^ and the nonpregnant state, such as abnormal uterine bleeding, postmenstrual spotting.^[10]^

Uterine niche, also called cesarean scar defect, deficient cesarean scar, pouch, or diverticulum, is defined as a triangular anechoic structure at the site of the scar or a gap in the myometrium at the site of a previous cesarean section.^[11]^ It is one of the most common complications associated with previous cesarean section. The diagnosis of uterine niche can be made by transvaginal ultrasonography,^[12]^ and the best time to perform ultrasonography is in the early proliferative phase.^[13]^ The main clinical manifestations are postmenstrual spotting and intrauterine infection, which may seriously affect the daily life of nonpregnant women.^[14]^ The etiopathogenesis of the uterine niche may be a result of suturing technique during previous cesarean section^[15]^ as well as patient-related factors, such as maternal age, number of previously performed cesarean sections, and flexion of the uterus.^[16]^ The incidence of uterine niche has been reported to be 78% in women with a history of 1 cesarean delivery.^[17]^ The treatment includes medical treatment, such as oral contraceptives,^[18]^ and surgical methods, such as hysteroscopy resectoscopic correction,^[19]^ endometrial ablation,^[20]^ laparoscopic surgery,^[13,21]^ and transvaginal repair surgery.^[22,23]^ Although good outcomes are reported in each study, present treatments could not decrease the incidence among women after undergoing cesarean section.

Smooth muscle cells (SMCs) are found within the walls of blood vessels, and organs such as the uterus and urinary bladder.^[24]^ Mesenchymal stem cells (MSCs) are long-lived cells with the ability of both self-renewal and differentiation into multipotential cells, such as osteoblasts, adipocytes, and SMCs.^[25]^ Human MSCs (hMSCs) can be isolated from somatic tissues, such as bone marrow, adipose or fetal tissue, including amniotic fluid, Wharton jelly, umbilical cord, and umbilical cord blood.^[26]^ Recent preclinical studies have found that MSCs play a major role in regulating SMC expression in animal model^[27,28]^ and exert a specialized function in arterial smooth muscle regeneration and functional recovery.^[29]^ Trials have shown an excellent safety and efficacy for the potential of MSCs as a therapeutic option for myocardial infraction.^[30–32]^

Cesarean uterine scar reconstruction is still a tough task for obstetricians and gynecologists. We postulate that MSCs can promote uterine scar reconstruction and reduce uterine niche incidence during cesarean section. To test the hypothesis, we therefore undertake a Phase II clinical trial of the treatment for uterine niche among primiparous women who undergo cesarean section.

## 2 Methods

**Figure 1:**
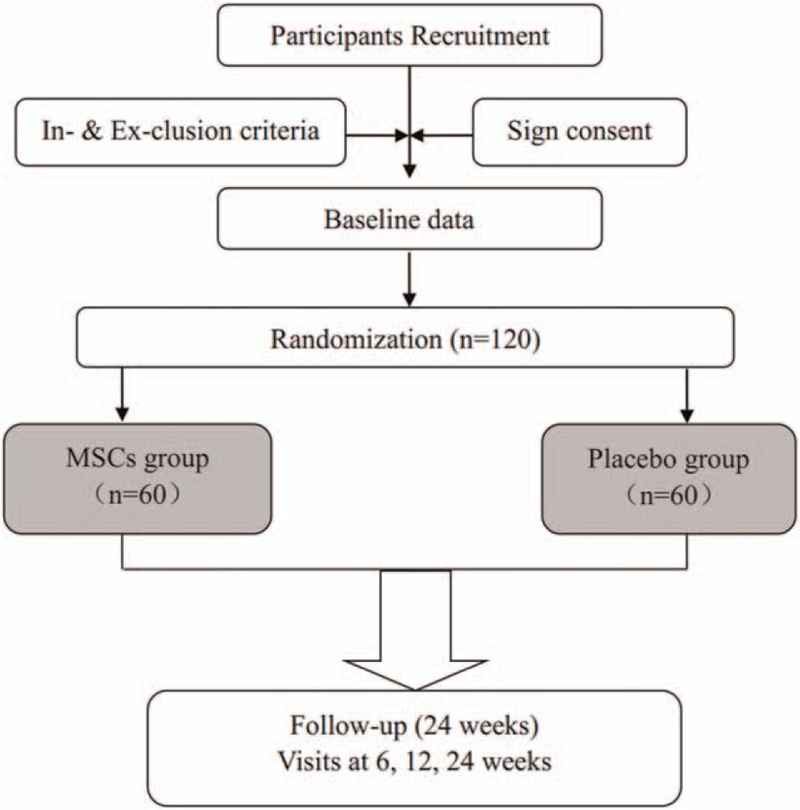
Chart of trial design.

This phase II clinical trial is a single-center, prospective, randomized, double-blind, placebo-controlled with 2 arms. The protocol has been developed following the Standard Protocol Items: Recommendations for Interventional Trials (SPIRIT).^[33]^ It will be conducted in accordance with the Declaration of Helsinki and all of the enrolled participants will be provided written informed consent. The protocol and the consent form have been approved by the ethics committee of the Southern Medical University Affiliated Maternal & Child Health Hospital of Foshan. All the randomization, allocation, recruitment, intervention, and analysis, etc., will be conducted in Southern Medical University Affiliated Maternal & Child health Hospital of Foshan. The overall trial profile is shown in Fig. 1. It has been registered with ClinicalTrials.gov, NCT02968459 (http://clinicaltrials.gov/).

### 2.1 Participants

Primiparous women with a singleton pregnancy between the 37th and 42nd week of gestation will be eligible for inclusion if they are aged 21 to 35 years. Gestational age will be estimated in accordance with the guidelines of the American College of Obstetricians and Gynecologists.^[34]^ Participants will come from our maternity clinic. Obstetricians will answer for the information, details, and requirements of the trial. We will exclude participants who met one of the following criteria: multiple gestation, placenta abruption, placenta previa, fibroids, antepartum hemorrhage, preeclampsia/eclampsia, hepatic or renal dysfunction, any systemic uncontrolled disease or inability to provide consent. After being screened for inclusion and exclusion criteria (Table 1), eligible participants will be introduced in the trial. Each eligible participant must provide their informed consent, and then randomly allocated to MSCs or placebo group according to a randomization numbers. Through the interview, the assistant researchers will be responsible to assess and record the participants’ baseline data.

**Table 1:**
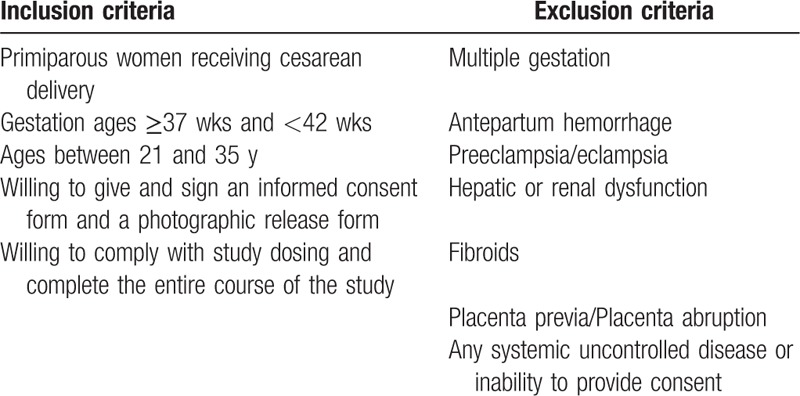
Eligibility criteria.

### 2.2 Randomization and masking

In total, 120 participants will be randomly assigned to 1 of 2 groups in a 1:1 ratio, the MSCs or placebo-controlled groups. Randomization numbers are completed by using the computer-generated list of random numbers. An independent statistician who is involved in neither the treatment nor data collection will randomize assignments in this study. It will be blinded to random allocation of participants to the 2 groups. The final group assignments will be sealed in opaque envelopes. Other than the investigational pharmacist who prepared the study drug, clinical and research staff members will be blinded in this trial. The assessors and statisticians for the data collection and analysis will be also blinded to the assignments.

### 2.3 Procedures

On the basis of previous preclinical safety and bioactivity analyses, participants will be randomly assigned to receive direct intramuscular injection of MSCs (a dose of 1∗10^7^ cells in 1 mL of 0.9% saline) (MSCs group) or an identical-appearing 1 mL of 0.9% saline (placebo-controlled group).

**Figure 2:**
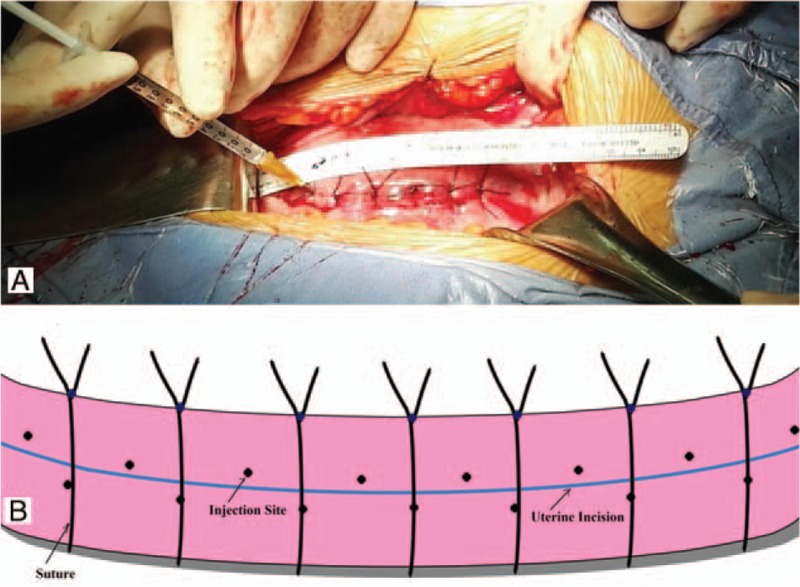
Direct intramuscular injection sites. (A) Real scene showing injection sites; (B) Schematic drawing showing injection sites. Injection sites will be selected near the incision at evenly 20 different sites.

Cesarean procedures and care followed usual practices. All participants will be performed by obstetricians from our department using a unified double-layer uterine closure technique with a continuous absorbable polyglycolic 1–0 suture. After suturing the uterine incision, direct local intramuscular injection will be performed in the uterine incision as soon as possible on the operating table. One milliliter will be injected as 20 aliquots of 0.05 mL into each injection site on the incision using 23-gauge needles. Injection sites will be selected near the incision at evenly 20 different sites (Fig. 2).

The MSCs are obtained from the umbilical cord of a healthy donor after delivery. All donors must be provided written informed consent. All procedures for cell processing are carried out according to Good Manufacturing Practice from Health-Biotech Pharmaceutical Company (Guangzhou, China).

MSCs release criteria for clinical use will be as follows: absence of pathogen contamination (bacterial, fungal, viral, or mycoplasma); viability >80% as evaluated by trypan blue staining; immune phenotype, showing the expression of CD73, CD90, and CD105 (Src homology-2) surface molecules (95%); and absence of CD45, CD34, CD11b, CD19, and HLA-DR markers (<2%).

### 2.4 Outcome

#### 2.4.1 Primary outcome

As a primary outcome of this clinical trial, we will evaluate the proportion of participants at 6 months who is found uterine niche in the uterus by transvaginal utrasonography.

**Figure 3:**
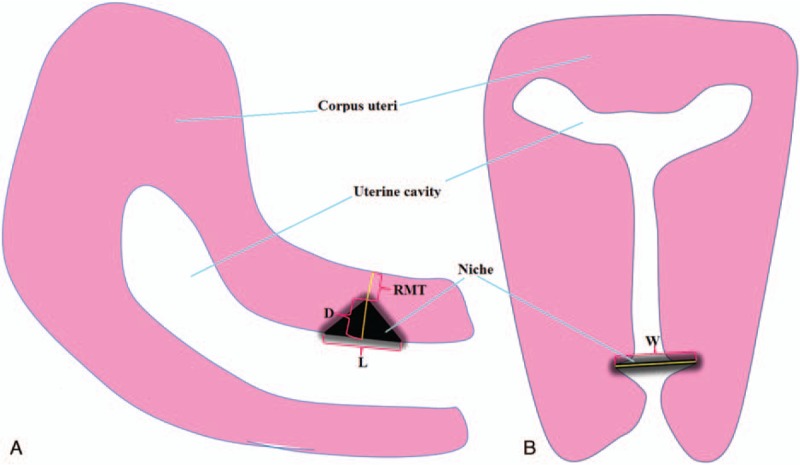
Schematic drawing showing niche parameters. (A) Longitudinal section of the uterus; (B) transverse section of the uterus. D = depth of the triangular hypoechoic scar niche; L = length of the triangular hypoechoic scar niche; RMT = residual myometrial thickness; W = width of the triangular hypoechoic scar niche.

The ultrasound examination will be performed using a 4 to 9 MHz convex transvaginal probe of Voluson E8 (General Electric Medical Systems, Chicago, IL). The niche is defined as a triangular anechoic area at the presumed site of incision.^[35]^ The standardized approach for imaging and measuring niche will be used as described in detail in a previous publication.^[12]^ Meanwhile, the following niche parameters, such as width of the triangular hypoechoic scar niche (W), depth of the triangular hypoechoic scar niche (D), length of the triangular hypoechoic scar niche (L), residual myometrial thickness (RMT), on the transection of the uterus will be also measured (Fig. 3). When the niche is not found, only the RMT value will be measured.

#### 2.4.2 Secondary outcome

Secondary outcomes will be measured individually at 6 weeks and 3 and 6 months post treatment. Table 2 has provided the time points and specific measurements of data. The mainly secondary outcomes are as follows:

(1) Uterine scar thickness and area
  The uterine scar thickness and area will be measured using a transvaginal utrasonography device.
(2) Mother's milk and serum will be collected at each visit and the immunoglobulin (IgG, IgA, IgM) and the complement (C3, C4) will be detected by transmission immune turbidity method using automatic biochemical analyzer.

**Table 2:**
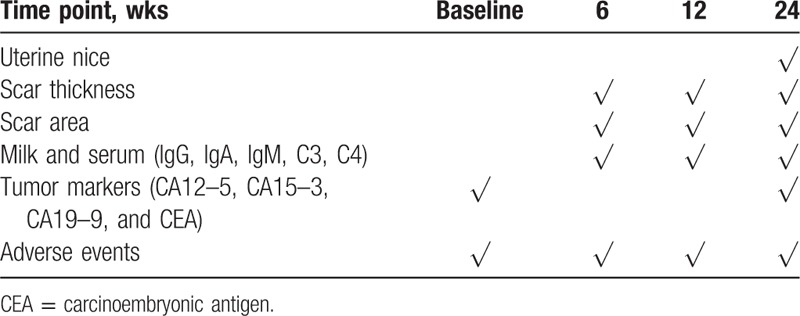
Schedule of visits and assessments.

### 2.5 Adverse events

On the basis of clinical severity, all adverse events, including mild, moderate, and severe, will be obtained and recorded at baseline and throughout the 6-month follow-up. Every time when follow-up, the participants will come to the interviewing room where the case report form (CRF) with adverse events will be filled with their feedback and the specialist checks. We will focus on the wound infection, endometritis, or other infections occurring up to 6 months after delivery in both groups. To evaluate tumor formation as a delayed reaction, we will test the tumor markers (CA12–5, CA15–3, CA19–9, and carcinoembryonic antigen) at baseline and 6 months. The principal investigator (ZL) will be responsible for managing the adverse events clinically. All adverse events will be reported to the Adverse Events Monitoring Committee.

### 2.6 Sample size

The sample size calculation is based on the primary outcome. According to previously published study, the proportion of uterine niche is 78% in women with a history of 1 cesarean delivery.^[17]^ We estimated a power of 90% and a 2-sided 0.05 level of significance, and a drop-out rate of 15% is taken into consideration. Finally, we plan to recruit 120 participants (60 in each group) would detect a 15% relative reduction in the primary outcome.

### 2.7 Data management and monitoring

All researchers will be given complete good clinical practical training. They will independently assess the effects of the interventions and collect the data. Both paper and electronic versions of the CRF will be kept in the secure research archives at the Southern Medical University Affiliated Maternal & Child Health Hospital of Foshan for 10 years. The principal investigator will be responsible for the confidential clinical information. Data monitoring committee, which comprises an epidemiologist, a statistician, and an obstetrician, will responsible for reviewing the results. They have the right to decide whether the trial should continue.

### 2.8 Statistical analysis

For data analysis, all analyses will be performed according to the intention-to-treat principle. Continuous data will be expressed as mean/standard deviation if it meets normal distribution, otherwise it will be presented by medians/interquartile range; categorical data will be expressed as frequency and percentage. In terms of comparison between the 2 groups, 2-tail Student *t* test for continuous data or the Chi-square test or Fisher exact test for categorical data will be used for outcomes. Relative risks and 95% confidence intervals will be calculated for outcomes. To assess changes of outcome levels over time, an analysis of variance (ANOVA) for repeated measures with post-hoc paired *t* tests or Wilcoxon signed rank test will be used for continuous data. Statistical significance is established at *P* < .05. Statistical analysis will be performed using R 3.0.3.

### 2.9 Trial status

The preparation of the study started in December 2016 and the first recruitment started in January 2017. It is anticipated to take 30 months to finish recruiting all the subjects and data collection. Data analysis is expected to be finished a month after data collection. The results will be disseminated via both national and international conferences and formal publication in an academic peer-reviewed journal.

## 3 Discussion

During the past decade, many experiments have demonstrated that MSCs have the characteristics of self-renewal and are able to differentiate into adipocytes, osteoblasts, neurons, myoblasts, and endothelium. They have been discovered in muscles, umbilical cord blood, amniotic fluid, and other tissues,^[36]^ and are potent therapeutic sources to repair damaged tissues, such as cardiac tissue, spinal cord injury, and complex perianal fistulas.

Limited clinical trials evidences have supported that MSCs is a safe and effective for refractory disease. A phase I, first-in-man study showed that single intracerebral doses of human neural stem cells were associated with improved neurological function and induced no adverse events.^[37]^ Another phase 3 trials also showed that allogeneic adipose-derived MSCs represented a safe and effective treatment for complex perianal fistulas in patients with refractory Crohn disease.^[38]^ Meanwhile, a pilot trial suggested that autologous MSCs can be safely administered to the hearts with myocardial ischemia and impaired cardiac function.^[39]^ Furthermore, a randomized phase I/II clinical trial demonstrated that intradermal transplantation of autologous stem cells was an effective and safe strategy to promote in vivo mechanical stretch induced skin regeneration, and they can provide complex skin defect reconstruction with plentiful of tissue.^[40]^

Uterine niche, a major public health focus, is a common complication affecting about three-fourths women with previous cesarean section. Most women suffered the postmenstrual spotting and prolonged menstrual bleeding. In this prospective, randomized, double-blind, placebo-controlled with 2 arms study, we hypothesized that MSCs can promote uterine scar reconstruction and reduce uterine niche incidence during cesarean section.

To our knowledge, this trial is the first to investigate the potential of therapeutic use of MSCs for the management of uterine niche after cesarean delivery. The outcomes from this study will help to determine the efficacy and safety of MSCs treatment in uterine niche and bridge the gap with regards to the current preclinical and clinical evidence. Importantly, if a positive outcome is identified, it will provide us with an effective therapeutic strategy to combat uterine niche, and would pave the way for achieving enormous public health benefit.
